# CRISPR-Cas Adaptive Immune Systems of the Sulfolobales: Unravelling Their Complexity and Diversity

**DOI:** 10.3390/life5010783

**Published:** 2015-03-10

**Authors:** Roger A. Garrett, Shiraz A. Shah, Susanne Erdmann, Guannan Liu, Marzieh Mousaei, Carlos León-Sobrino, Wenfang Peng, Soley Gudbergsdottir, Ling Deng, Gisle Vestergaard, Xu Peng, Qunxin She

**Affiliations:** 1 Archaea Centre, Department of Biology, Copenhagen University, Ole Maaløes Vej 5, DK2200 Copenhagen N, Denmark; E-Mails: sashah@bio.ku.dk (S.A.S.); liuguannan@bio.ku.dk (G.L.); mmousaei@bio.ku.dk (M.M.); clsobrino@bio.ku.dk (C.L.-S.); wenfang.peng@bio.ku.dk (W.P.); soley.gudbergsdottir@bio.ku.dk (S.G.); lingd@bio.ku.dk (L.D.); peng@bio.ku.dk (X.P.); qunxin@bio.ku.dk (Q.S.); 2 School of Biotechnology and Biomolecular Sciences, University of New South Wales, 2052 Sydney NSW, Australia; E-Mail: s.erdmann@unsw.edu.au; 3 Helmholtz Zentrum München, Research Unit Environmental Genomics, Ingolstädter Landstraße 1, 85764 Oberschleißheim, Germany; E-Mail: gisle.vestergaard@helmholtz-muenchen.de

**Keywords:** CRISPR-Cas, immune response, crenarchaea, archaeal viruses, conjugative plasmids, adaptation, interference, crRNA, integration, transposition

## Abstract

The Sulfolobales have provided good model organisms for studying CRISPR-Cas systems of the crenarchaeal kingdom of the archaea. These organisms are infected by a wide range of exceptional archaea-specific viruses and conjugative plasmids, and their CRISPR-Cas systems generally exhibit extensive structural and functional diversity. They carry large and multiple CRISPR loci and often multiple copies of diverse Type I and Type III interference modules as well as more homogeneous adaptation modules. These acidothermophilic organisms have recently provided seminal insights into both the adaptation process, the diverse modes of interference, and their modes of regulation. The functions of the adaptation and interference modules tend to be loosely coupled and the stringency of the crRNA-DNA sequence matching during DNA interference is relatively low, in contrast to some more streamlined CRISPR-Cas systems of bacteria. Despite this, there is evidence for a complex and differential regulation of expression of the diverse functional modules in response to viral infection. Recent work also supports critical roles for non-core Cas proteins, especially during Type III-directed interference, and this is consistent with these proteins tending to coevolve with core Cas proteins. Various novel aspects of CRISPR-Cas systems of the Sulfolobales are considered including an alternative spacer acquisition mechanism, reversible spacer acquisition, the formation and significance of antisense CRISPR RNAs, and a novel mechanism for avoidance of CRISPR-Cas defense. Finally, questions regarding the basis for the complexity, diversity, and apparent redundancy, of the intracellular CRISPR-Cas systems are discussed.

## 1. Introduction

In Copenhagen, our first insights into archaeal CRISPR (clustered regularly interspaced short palindromic repeats)-Cas (CRISPR-associated) systems occurred while sequencing the genome of the hyperthermophilic crenarchaeon *Sulfolobus solfataricus* P2 [1]. This strain, together with the closely related strain P1, had been widely used as preferred hosts for propagating several novel archaeal viruses and plasmids by Wolfram Zillig and colleagues [2]. The genome sequencing project, extending over the period 1997–2000, revealed large regions of regularly interspaced direct repeats, with a total of 421 repeats and encompassing about 30 kbp of DNA [1]. Later work showed that *S. solfataricus*, and other *Sulfolobus* species, carried both extended repeat clusters and multiple and diverse Cas gene cassettes which later became implicated in the spacer acquisition and nucleic acid interference stages of the CRISPR-Cas adaptive immune response, reviewed in [3,4]. Although these *Sulfolobus* systems were found to be more complex than those present in most bacteria, they gradually proved tractable to experimental study aided, in large part, by the development of a range of robust genetic systems for these acidothermophilic archaea [5,6,7,8,9,10,11,12].

The seminal breakthrough in determining the primary function of the CRISPR loci in archaea and bacteria arose from the demonstration by Mojica *et al.* [13], Pourcel *et al.* [14] and Bolotin *et al.* [15] that many of the spacers located between the repeats of different organisms were likely to derive from invasive genetic elements, primarily viruses. It was also demonstrated that CRISPR arrays were widespread in both archaea and bacteria [13].

## 2. Viruses and Conjugative Plasmids of the Sulfolobales

### 2.1. Viruses

Electron micrographs of environmental isolates from solfataric fields have revealed numerous diverse virus-like particles some of which remain uncharacterised [2]. Isolated viruses known to infect members of the Sulfolobales are summarised in Table 1. They show a variety of morphotypes and carry circular or linear dsDNA genomes and they have been classified on the basis of these properties into several viral families [16,17] which are archaea specific [18,19]. Genomic fragments of positive strand RNA viruses which may infect archaea have also been detected in acidic hot springs rich in archaeal hyperthermophiles [20].

**Table 1:** Summary of viruses infecting the Sulfolobales. All viral genome sequences are available from the EBI database: https://www.ebi.ac.uk/genomes/archaealvirus.html. The sequences for ATV2, SIRV4 and SIFV2 remain unpublished [21].

Family	Name	Host	Morphotype	Genome	Integrates	Genome Size
*Fuselloviridae*	ASV1	*Acidianus*	Fusiform	Circular	yes	24,655
	SSV1, SSV2,	*Sulfolobus*				15,465, 14,796
	SSV4, SSV5,	"				15,135, 15,330
	SSV6, SSV7	"				15,684, 17,602
	SMF1	*Sulfolobales*				14,847
Icosahedral	STIV	*Sulfolobus*	Turreted icosahedral	Circular	no	17,663
	STIV2	"				16,622
*Bicaudaviridae*	ATV, ATV2	*Acidianus*	Tailed-fusiform	Circular	yes	62,730, 57,909
	SMV1	*Sulfolobus*				48,775
Monocauda-viruses	STSV1	*Sulfolobus*	Tailed-fusiform	Circular	yes	75,294
	STSV2	"				76,107
*Rudiviridae*	ARV1	*Acidianus*	Rod-shaped	Linear	no	24,655
	SIRV1, SIRV2	*Sulfolobus*				32,308, 35,450
	SIRV4	"				32,992
	SMR1	*Sulfolobales*				27,431
*Lipothrixviridae*	AFV1, AFV2	*Acidianus*	Filamentous	Linear	no	20,869, 31,787
	AFV3, AFV6	"				40,449, 39,577
	AFV7, AFV8	"				36,895, 38,179
	AFV9	"				41,172
	SIFV, SIFV2	*Sulfolobus*				40,900, 39,399
*Ampullaviridae*	ABV	*Acidianus*	Bottle-shaped	Linear	no	23,814
*Guttaviridae*	SNDV	*Sulfolobus*	Bearded droplet	Circular(modified)	-	unsequenced

### 2.2. Conjugative Plasmids

Conjugative plasmids, specific to the Sulfolobales, show extensive similarity in their genomes and are listed in Table 2. All except pHVE14, pMGB1, pAH1 and pTC were first characterised by Zillig and coworkers [2]. They encode a cluster of five to six core proteins that have been implicated in the conjugative process and they all encode an integrase, except for pTC which may have derived from an integrase-encoding plasmid [22,23,24,25,26,27,28]. Although the plasmids tend to propagate stably in their original host strains, when transferred to laboratory *S. solfataricus* strains they often generate mixtures of variant plasmids and some of these variants are formed by recombination between characteristic sequence motifs distributed around the genomes [25,26]. Moreover, plasmids pNOB8 and pKEF9 carry small CRISPR loci, with virus-matching spacers, suggesting a potential antagonistic role towards coinfecting viruses [23,26,29]. Both intact and degenerate forms of similar plasmids are often found integrated at tRNA genes of host genomes [1,30].

**Table 2:** Conjugative plasmids isolated from the Sulfolobales. Genome sequences are available at: http://www.ebi.ac.uk/genomes/plasmid.html.

Plasmid	Host	Origin	Genome size
pARN3	*S. islandicus*	Iceland	26,200
pARN4	"	"	26,476
pHVE14	"	"	35,422
pING1	"	"	24,554
pKEF9	"	"	28,930
pSOG1	"	"	29,000
pSOG2	"	"	25,960
pAH1	*Acidianus hospitalis*	Italy	28,649
pMGB1	*S. solfataricus*	USA	27,975
pNOB8	*Sulfolobus* sp. NOB8H2	Japan	41,229
pTC	*S. tengchongensis*	China	20,417

## 3. Different Classes of CRISPR-Cas Systems

### 3.1. Structural Classification

Archaeal CRISPR-Cas systems have been classified into two major classes, Type I and Type III, based primarily on their Cas protein contents and amino acid sequences [31]. Archaea lack the RNase III enzyme that is essential for CRISPR transcript processing in the bacteria-specific Type II system [32,33,34]. Type I and Type III systems have each been divided into multiple subtypes that are quite diverse with respect to the protein components of their interference complexes [35]. These subtype classifications were recently reexamined for archaea using an altered strategy from that used earlier [31] and based primarily on gene content and synteny and concatenated Cas protein sequences of the interference modules [36]. This choice was made because separate dendrograms based on archaeal adaptation or interference modules yielded different branching patterns consistent with the occurrence of modular exchange between the CRISPR-Cas systems [37,38,39]. Evidence for a similar exchange of adaptation and interference modules has also been presented recently for a bacterial Type I-E system [40]. For the Sulfolobales, this modular exchange is consistent with the independent regulation of the two modules by different Csa3 proteins (Section 7.1).

**Figure 1:**
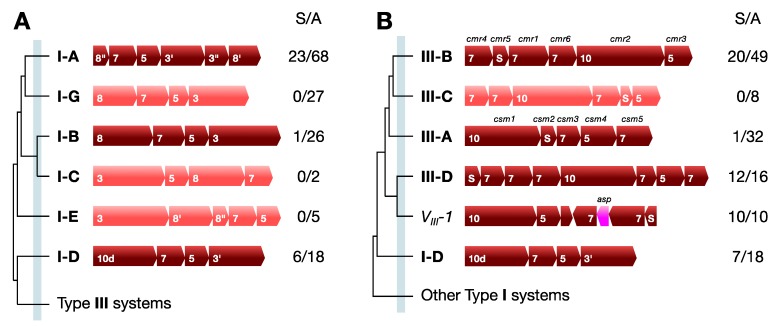
Dendrograms of archaeal CRISPR-Cas interference gene cassettes. (**A**) Type I and (**B**) Type III, where gene identities, sizes and syntenies are shown for representatives of the different subtypes. Total numbers of identified subtypes are indicated on the right for Sulfolobales (S) and all archaea (A). Standard *csm/cmr* gene names are given for subtypes III-A and III-B. Different subtypes have distinct gene syntenies and branch before the defined threshold indicated by the light blue vertical line defined earlier [36]. Subtypes III-C and III-D correspond to the earlier defined families A and D [42] while variant subtype V_III_-1 is only found in some members of the Sulfolobales [36]. All Type III gene cassettes carry *cas10*, the gene for protein S, *cas5*, and multiple *cas7* paralogues. *asp* denotes the putative aspartate protease gene. The subtype I-D gene cassette branches at the junction of the Type I and Type III subtypes (based on data in [36]).

For Type I systems, reevaluation of archaeal subtype classifications based on interference complexes, led to the proposal to divide subtype I-B into subtypes I-B and I-G, which are similar to the earlier proposed groupings Hmar and Tneap, respectively (Figure 1A) [41]. Moreover, previously, we had proposed dividing archaeal Type III systems into five families A to E [42]. Later family E became Type III-A and families B and C became Type III-B and, to conform with this widely used nomenclature, families A and D became subtypes III-C and III-D, respectively (Figure 1B) [36]. The most common archaeal Type I subtypes are I-A, I-B and I-D and I-G while subtypes I-C and I-E occur rarely and subtype I-F has not been detected [36]. For the archaeal Type III systems, subtypes III-A and III-B dominate and III-C and III-D are less common. In addition, numerous variant subtypes have been identified some of which are phyla specific and their number may increase as more genomes are sequenced. Among the Sulfolobales, subtypes I-A, I-D, III-B and III-D dominate together with a single Type III variant V_III_-I that is exclusive to the Sulfolobales [36] (Figure 1).

Although basic differences have been found between the interference targets of the Type I and Type III systems (Section 6.3 and Section 6.4), at present less is known about how the structural differences of the subtypes influence their modes of interference (see below).

### 3.2. Functional Classification

Some progress has been made in defining structure-function relationships of the different CRISPR-Cas subtypes. Clearly, there are a limited number of possible interference targets including dsDNA, ssDNA, replicating and transcribing DNA, transcripts and, possibly, viral ssRNA or dsRNA. However, this list could be extended if, for example, nucleic acids carrying specific chemical modifications can be selectively targeted by specialised CRISPR-Cas interference complexes. Currently, there is a consensus that most Type I systems, and bacterial Type II systems, target dsDNA, while evidence from *in vitro* and *in vivo* experiments support Type III-B systems targeting RNA [43,44,45,46].

Unexpectedly, it was shown that the two Type III-B systems in *Sulfolobus islandicus* REY15A, denoted Cmr-α and Cmr-β, interfere by different mechanisms. [47,48]. The Cmr-β complex, consistent with the above-mentioned results, cleaves transcripts relatively efficiently, probably by recycling. In contrast, the Cmr-α complex, in the presence of a non-core Csx1 protein, cleaves both RNA and transcribing DNA. This result suggested that complexing with a *de novo* transcript facilitates identification of the DNA target [48] (Section 6.5). Targeting of transcribing DNA has also been observed for a bacterial Type III-A system although it remains unclear whether accessory Cas proteins were involved [49]. However, the *Sulfolobus* results underline that the functionally diverse non-core Cas proteins, primarily linked genetically to Type III systems, can influence their interference mechanisms [36,38,50] (Section 9).

## 4. Properties of CRISPR Loci

### 4.1. Repeats, Spacers and Leaders

**Figure 2:**
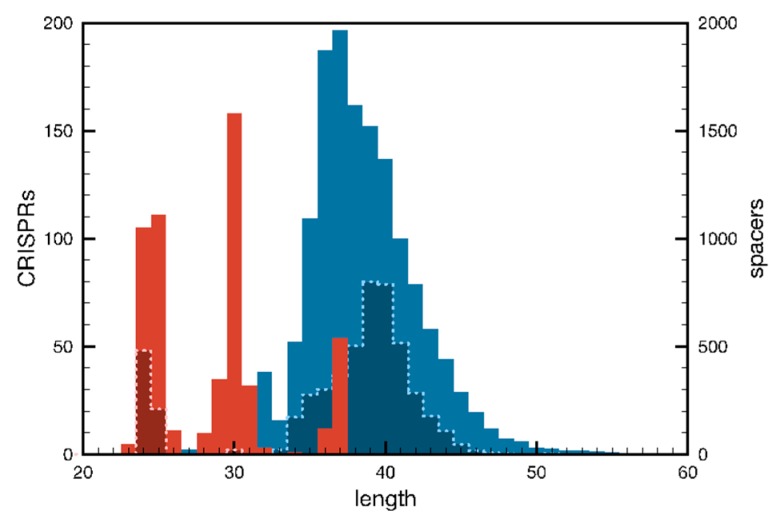
Histogram showing the distribution of sizes of repeats (red) and spacers (blue) for all archaeal CRISPRs [36]. Size distributions for repeats and spacers of the Sulfolobales are indicated by striated lines.

**Figure 3:**
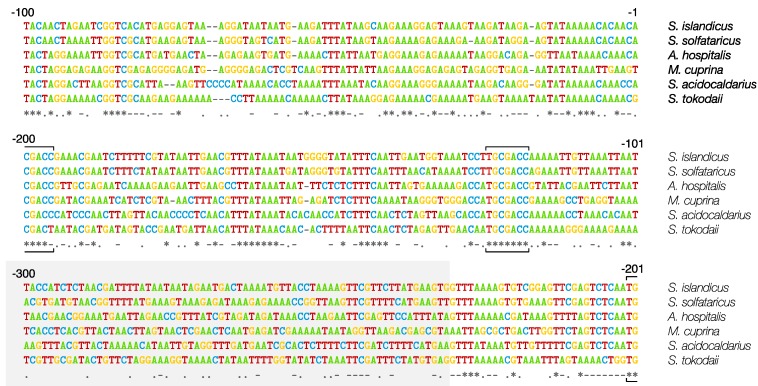
Alignment of leaders from CRISPR loci (Type I-A, PAM-CCN) of diverse members of the Sulfolobales showing significant levels of sequence identity over the first 230 bp from repeat 1, after which shared identity decreases (shaded area). Conserved sequence positions are indicated by asterisks and conserved sequence motifs are bracketed. The archaeal genome sequences are available at: https://www.ebi.ac.uk/genomes/archaea.html.

CRISPR loci consist of contiguous repeat-spacer units where the repeat is generally invariant, in size and sequence, for a given CRISPR locus whereas spacers usually all differ in sequence, and in length. Archaeal repeats fall into three size groups of about 24, 30 and 37 bp (Figure 2), differing by a little over half a turn of a DNA double helix, which may have some mechanistic significance for the adaptation, processing or interference reactions. Repeats of the Sulfolobales and many other crenarchaea are generally 24–25 bp while spacers lie mainly in the size range of 35–43 bp (Figure 2). Most CRISPR loci are preceded by leaders of approximately 200–400 bp which carry some low complexity sequence regions. An alignment of leader regions of Type I-A CRISPR loci (subfamily 1, see below) from diverse members of the Sulfolobales is shown in Figure 3. There is a significant degree of sequence conservation, including a few conserved motifs, for about 230 bp beyond the first repeat, after which sequence similarity gradually decreases.

For the Sulfolobales, dendrograms based on sequence alignments were generated for Cas1, and CRISPR repeats and leaders, and they provided support for three main subfamilies of Type I-A systems. Each carried a consensus predicted protospacer adjacent motif (PAM) sequence; subfamily 1-CCN, subfamily 2-TCN and subfamily 3-GTN [37,38,39]. This result was consistent with the coevolution of Cas1, repeats, leaders and PAM sequences and their mutual involvement in adaptation. However, different dendrograms were obtained from concatenated protein sequences of the interference modules consistent with their capacity to exchange their adaptation module partners [39,40].

Such a bioinformatical analysis cannot be extended more widely for leaders because of their variable sizes and limited sequence conservation. However, a dendrogram of 3500 repeats of archaea and bacteria, based on sequences and structural properties, demonstrated that the repeats fall into six major clusters. Moreover, 96% of the crenarchaeal repeats fall within a single cluster F consistent with their confinement to the Crenarchaeota [51,52].

### 4.2. Spacer Sequence Matches to Invasive Genetic Elements

**Figure 4:**
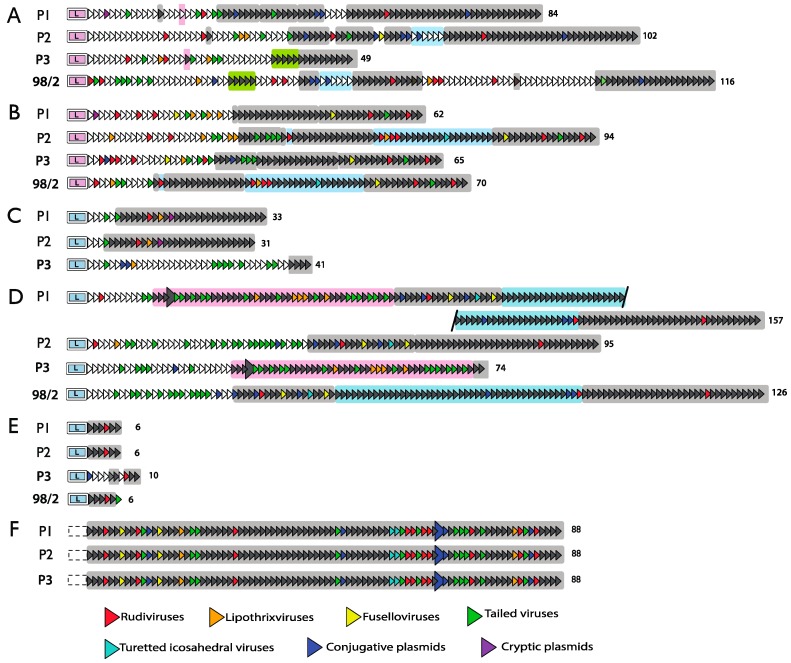
Comparison of CRISPR loci of *S. solfataricus* strains P1, P2, P3 and 98/2. Spacers are coloured to identify virus and plasmid sequences with the best match. A significant match was defined as a maximum of 10 mismatches with no more than two in the critical annealing region from positions 3–7 (Section 6.2). Each locus is oriented with the leader on the left. Colour-coding of virus and plasmid matches is indicated. The large arrowheads in loci D and F represent irregularities in the spacer-repeat structures (Section 4.5). Regions shaded in specific colours are identical in sequence. In the original publication of Lillestøl *et al.* [29], some parts of the CRISPR arrays were inadvertently inverted relative to the leader [57] and were later corrected in [37].

Bioinformatical analyses of CRISPR loci of the Sulfolobales revealed many potentially significant spacer sequence matches to viruses or conjugative plasmids and they were identified earlier for the large CRISPR loci of *S. solfataricus* P1 and P2 and different *S. islandicus* strains [29,37,53,54,55,56]. The *S. solfataricus* matches are presented here (Figure 4) together with those of the more recently sequenced CRISPR loci of strains P3 and 98/2 employing more rigorous stringency criteria than used earlier.

Strain P3 was isolated about 30 years after strains P1 and P2 from the same solfataric area near Naples and it exhibits large conserved, or deleted, segments in CRISPR loci A, B, C and D [21]. In contrast, locus F which lacks a leader is completely conserved in spacer content and sequence, although it is absent from strain 98/2 which may have originated from the geographically distant Yellowstone National Park (Figure 4).

A puzzle arising from seminal spacer matching analyses was the high number of significant matches (about 30% in *S. solfataricus* strains) to the relatively few viruses and plasmids that had been sequenced [37,53,56]. This may reflect a tendency for sequenced genetic elements to predominate in geographic locations from which the host genomes were isolated and sequenced. A supposition that receives support from studies showing the presence of distinct fuselloviruses located in specific geothermal areas [54]. Moreover, Icelandic *S. islandicus* strains are rich in spacers matching rudiviral genomes [55,58] and their rod-shaped virions are commonly seen in electron micrographs of Icelandic environmental samples [2]. *S. solfataricus* strains and *Metallosphaera sedula* also carry many significant matches to the bicaudavirus ATV and all originate from the same solfataric area near Naples [37,59].

Pie plots were generated earlier for two major Type I-A subfamilies (utilising different PAM sequences) and a Type I-D system, found amongst the Sulfolobales (Section 4.1), to test whether there was a functional link between specific CRISPR loci and a given type of genetic element that was subjected to adaptation [37]. The relative numbers of significant spacer matches to different viral families of the Sulfolobales were estimated for rudiviruses, lipothrixviruses, fuselloviruses, a bicaudavirus, and conjugative and cryptic plasmids. Despite the different groups of CRISPR loci showing significant biases in spacer matches to the bicaudavirus, rudiviruses or conjugative plasmids, respectively, the Pie-plots exhibited fairly balanced patterns of spacer matching to different classes of genetic elements, and it was inferred that there was no strong bias of a CRISPR locus for a particular type of genetic element [37].

### 4.3. Transcription and Processing

Large CRISPR loci of *Sulfolobus* species are transcribed from promoters within the leaders and are processed within the repeats into small CRISPR RNAs (crRNAs) of about 50–60 nt [29,37]. The basic processing mechanism of CRISPR transcripts within the repeats was first elucidated during a transcriptome study of the euryarchaeon *Archaeoglobus fulgidus* [60] and later *S. solfataricus* P1 [61]. crRNAs carrying a single spacer were first detected from Northern analyses of RNA extracts from *Sulfolobus acidocaldarius* [29] and the mature crRNAs, that had undergone additional 3'-end processing associated with Type III-directed interference, were characterised in *S. islandicus* and *S. solfataricus* species [47,62,63]. All the mature crRNAs carry an 8 nt 5'-repeat tag.

In contrast to the Type I-E system of *Escherichia coli*, where the RNA processing enzyme CasE binds to the interference (CASCADE) complexes [64], Cas6, the corresponding enzyme in *Sulfolobus* species, can function alone [47]. It recognises the repeats of CRISPR transcripts and cleaves them at specific sequences *in vitro* [62]. Crystallographic studies have shown that a Cas6 protein of *S. solfataricus* forms an unusual dimeric structure that lacks the canonical catalytic histidine residue characteristic of other Cas6 proteins. It was inferred that this protein is multifunctional, exhibiting low catalytic activity, and additional crRNA recognition and chaperone-like properties [65]. *S. solfataricus* was also shown to carry two types of Cas6 protein, exhibiting altered specificities for different repeat sequences present in the multiple CRISPR loci of the host [66]. One of these could also process a non-coding RNA *in vivo* consistent with some Cas6 proteins recycling and performing alternative cellular functions [66]. Genetic studies of individual *cas* genes in *S. islandicus* have confirmed that only *cas6* inactivation abolished CRISPR transcript processing and they showed further that other Cas proteins, including Cas3, Cas5 and Cas7, were involved in post-cleavage maturation or stabilisation of crRNA in the Type I-A system of *S. islandicus* [63]. In the Type I-B system of *Haloferax volcanii*, the core Cas proteins Cas5, Cas6 and Cas7 of the interference complex were also shown to be necessary for crRNA maturation and stability [67].

It was always likely that internal transcription could be initiated within CRISPR loci, in sense or antisense directions, from archaeal promoters taken up randomly in spacer regions [68]. For the sense strand, it is unclear to what extent RNA polymerase accumulation at internal sites would interfere with primary CRISPR transcription and thereby influence the relative yields of different crRNAs. Antisense CRISPR transcripts were first observed from each of the multiple CRISPR loci of *S. acidocaldarius* by Northern blotting, although their yields were not quantified in relation to the overall expression levels of the CRISPR loci [29,37]. However, a transcriptome study of *S. solfataricus* P2 grown on different carbon sources revealed detailed insights into the yields and 5'-end sequences of many non-coding RNAs, including pre- and mature crRNAs [69]. Based on these data, specific promoter sites were identified within CRISPR spacers from which both sense and antisense transcripts were produced [70] (Section 7.6).

### 4.4. Structural Instability of CRISPR Loci

*Sulfolobus* CRISPR loci occur in a wide range of sizes often extending to over 100 spacer-repeat units per locus and preceded by leaders of up to a few hundred base pairs [29]. Locus sizes tend to increase periodically during adaptation reactions. For example, up to eight *de novo* spacers were incorporated into one CRISPR locus of virus-infected *S. islandicus* REY15A during an adaptation reaction [71,72]. Thus, other mechanisms are likely to operate to limit overall locus size. The alignment of related CRISPR loci of *S. solfataricus* P1 and P2 strains provided evidence for large internal deletions having occurred over time (Figure 4) [29,37]. This comparison revealed further that: (a) *de novo* spacers tend to accrue at the leader end of the locus, and (b) that spacer-repeat regions sometimes duplicate within CRISPR loci and can even recombine between different CRISPR loci intracellularly [29,37]. Exceptional was the leaderless 88 spacer CRISPR locus F of *S. solfataricus* that was inactive in spacer acquisition and invariant in spacer content and sequence (Figure 4).

Structural changes within CRISPR loci were inferred to occur via recombination between the direct repeats. Such a mechanism is consistent with the finding that recombination can occur between relatively short repeat sequences in *S. acidocaldarius* [73]. The recombination events are likely to be essentially random, although they may be favoured for repeat pairs where sequence matches extend into adjacent spacer regions. Although some CRISPR spacer heterogeneity within a population is likely to be advantageous in promoting and maintaining cellular diversity [74], there must be a selection process operating after the adaptation stage, if one assumes that the enormous variety of diverse *de novo* spacers observed in laboratory adaptation experiments are replicated in similar events occurring in solfataric fields [72,75,76]. For example, there may be a selection against cells carrying several *de novo* spacers once the genetic element has been purged from the culture, or against *de novo* spacers with significant sequence matches to the host chromosome [77], or against those carrying promoter or terminator motifs that can interfere with primary CRISPR transcription [70]. Given that individual crRNAs can be utilised by both Type I and Type III interference complexes, it is unlikely that the specific interference target directly influences which *de novo* spacers are retained [47,48] (Section 6.5).

CRISPR deletions may be favoured if, for example, an invasive element carries a spacer matching sequence and one or more genes that are advantageous for host survival. For two uracil auxotrophic *Sulfolobus* strains, transformation of such a plasmid carrying *pyrE/pyrF* genes led to the loss of CRISPR segments carrying the matching spacer [70,78]. While it is likely that most of these deletions occurred by random recombination between repeats, in 50% of surviving transformants of *S. islandicus* REY15A, the single matching spacer was selectively deleted which raised the possibility of the operation of a reverse spacer acquisition mechanism [78] (Section 5.7).

### 4.5. Integrity of the Spacer-Repeat Substructure

Very few structural irregularities are observed in CRISPR loci and the spacer-repeat unit length seems to be maintained, despite the small variation in length of the spacers [29]. For example, in locus D of *S. solfataricus* P2, half a spacer is followed by two atypical repeats followed by a regular repeat-spacer unit, and in CRISPR locus-121 of *Sulfolobus tokodaii* two atypical repeats are 18 bp longer than the other repeats but are followed by shorter spacers. In both cases, the overall repeat-spacer unit size is maintained along the CRISPR locus. Moreover, the 899 bp fragment that partially matches a pNOB8-like conjugative plasmid inserted between two repeats in locus F of *S. solfataricus* [37] and corresponds approximately in size to 13 repeats and 14 spacers.

Although many *Sulfolobus* species, including *S. solfataricus*, carry a high level of diverse IS elements and MITEs, the CRISPR loci appear to be relatively intractable to transpositional events despite the presence of numerous potential target sites in the spacers [79,80,81]. In an early study, no mobile elements were detected in CRISPR loci of crenarchaeal genomes and only a single IS element (ISH4) and a 132 bp MITE were detected in euryarchaeal loci [29]. Moreover, amongst the Sulfolobales one rarely encounters evidence of CRISPR locus disruption resulting from recombination between similar IS elements [79], although CRISPR-Cas systems are sometimes bordered by IS elements [82].

An exception, which reinforces this rule in the sense that transpositional events are very rare, was observed in the experiment when host CRISPR spacers of uracil auxotrophic *S. solfataricus* were challenged by a plasmid carrying a spacer-matching sequence and maintained under selection (Section 4.4). Whereas in most surviving transformants the matching spacer was deleted, the remainder carried insertions of ISC1359 in the matching spacer which would have inactivated production of the mature crRNA and thereby prevented plasmid interference [78].

The structural invariance of the leaderless CRISPR locus F in *S. solfataricus* (Figure 4) raised the possibility that structural changes in CRISPR loci occur concurrently with *de novo* spacer acquisition because the spacer insertion involves Cas1-facilitated cleavage and repair of the CRISPR locus. This hypothesis receives some support from the observation that a clone of *S. islandicus* undergoing spacer acquisition from the virus STSV2 had incurred a major deletion in one of the active CRISPR loci [72]. The hypothesis is also consistent with evidence showing that Cas1 of *E. coli* (YgbT) physically and genetically interacts with major components of DNA repair systems [83].

## 5. Modular Mechanisms of Adaptation

### 5.1. Adaptation Module

The proteins implicated in adaptation, Cas1, Cas2, and generally Cas4 for the Sulfolobales, are encoded in separate gene cassettes from the interference proteins, and the two modules tend to evolve independently [36,38,39]. Adaptation gene cassettes are commonly located adjacent to a CRISPR locus for the predominantly Type I-A and I-D systems of the Sulfolobales whereas multiple copies of different Type III interference complexes are often co-encoded on the host genomes but are located distantly from CRISPR loci [36,55]. Consistent with these observations, it was demonstrated for *S. islandicus* REY15A, carrying a Type I-A and two different Type III-B interference complexes, that a single adaptation module recognises the PAM sequence CCN and generates *de novo* spacers which, in turn, yield crRNAs processed by a single Cas6 ribonuclease [47].

In adaptation experiments with virus-infected *Sulfolobus* hosts, delays of up to 12 days occurred post infection (p.i.) before spacer acquisition was detected, and the activation coincided with a strong decrease in growth rate. Spacer uptake continued for several days with multiple spacers being inserted into different CRISPR loci [72,75]. For *S. solfataricus* P2 infected with a mixture of the virus SMV1 and conjugative plasmid pMGB1, spacer acquisition continued until day 16 p.i. when *de novo* spacer yields plateaued to yield a maximum of three and four pMGB1 spacers, respectively, in the two most active loci C and D [75]. When *S. islandicus* REY15A was infected with SMV1 + STSV2, spacer uptake in two CRISPR loci was monitored over a 70 day period. On average, there were 2.4 *de novo* spacers per clone at 20 days p.i., increasing to seven *de novo* spacers per clone at 30 days with a maximum of five and eight new spacers detected in CRISPR loci 1 and 2, respectively [72]. Thus, both the activation of spacer acquisition, and the process of spacer uptake, occur over several days resulting in the insertion of multiple *de novo* spacer into the CRISPR loci.

### 5.2. Mechanism of Protospacer Selection

CRISPR spacers vary in the size range 35–43 bp and are generated from invading genetic elements by either excision or, possibly, a copying mechanism. A detailed study of the locations of overlapping protospacers on pMGB1 that had yielded *de novo* spacers in *S. solfataricus*, led to the hypothesis that an imprecise molecular ruler mechanism operated measured from the PAM end of the protospacer [75].

There are invariably many potential protospacers available. For example, a total of 1546 CCN PAM sequences were identified in pMGB1, each of which can produce overlapping spacer sequences of differing lengths leading, potentially, to a few thousand unique *de novo* spacers. Consistent with this, CRISPR sequencing of many infected clones of *S. solfataricus* P2 yielded few duplications of the 409 unique *de novo* spacers inserted into CRISPR loci C, D and E [75,76].

Early statistical analyses of predicted CRISPR spacer matches on genomes of several viruses and plasmids of the Sulfolobales concluded that they were essentially randomly distributed [53,56]. More recently, analysis of *de novo* spacers obtained from pMGB1 and STSV2 undergoing adaptation in different *Sulfolobus* species reinforced a lack of significant bias in genomic locations, DNA strand direction, or gene *versus* intergenic regions, with one exception described below for the conjugative plasmid (Section 5.3). This lack of bias in genomic location or direction also extended to the multiple *de novo* spacers accrued within a given CRISPR locus in individual clones [72,75,76] (Section 5.6). These findings contrast with results obtained for a Type II CRISPR-Cas system of the lytic phage-infected *Streptococcus thermophilus* where a strong bias to five phage DNA regions was observed [84] and the results are difficult to reconcile with a "priming" mechanism for adaptation in *Sulfolobus* (Section 5.6).

### 5.3. Exceptional Biased Spacer Selection from a Conjugative Plasmid

At present, little is known about the mechanism of conjugative DNA transfer in the Sulfolobales. Only about six conserved plasmid proteins have been implicated in this process, including distant homologs of the bacterial TrbE and TraG proteins, and this lack of genetic complexity renders it likely that a dsDNA transfer mechanism is active [26].

Adaptation experiments with pMGB1 provided one exceptional example of biased protospacer selection [75,76]. For each experiment, a single *de novo* spacer was highly overrepresented (13%–29%) for each of the three most active CRISPR loci of *S. solfataricus* and, moreover, the same sequences were dominant in independent experiments [76]. One of these *de novo* spacers, in locus E, derives from the gene encoding ORF472, a highly conserved membrane protein, while those in loci C and D arose from DNA encoding the truncated C-terminal domain of a membrane transporter, ORF128, for which the N-terminal domain had been disrupted by a MITE insertion. Whereas the spacer-specific crRNA targeting ORF472 was dsDNA specific, both crRNAs matching ORF128 could cause either mRNA or DNA interference. Both ORFs are potential candidates for involvement in conjugative DNA transfer and the result suggests that these two DNA regions, and/or the transcript of ORF128, may initially have been strongly targeted [76].

### 5.4. De Novo Spacer Insertion

Early bioinformatic analyses indirectly implicated the leader region in the adaptation process [38] but the first experimental evidence was obtained for the genetically modified *E. coli* Type I-E system. The results indicated that the first 60 bp of the leader, and the first repeat, were critical for spacer acquisition [85]. Experimental results from Mojica and colleagues limited the important leader region of this Type I-E system to 42 bp, and also provided support for a ruler mechanism operating during *de novo* spacer insertion [86], possibly complementing the proposed ruler mechanism for protospacer selection [75]. Such a spacer insertion mechanism would also be consistent with the rigorous maintenance of the regular spacer-repeat structure of CRISPR loci (Section 4.5).

For Type I systems, the PAM end of the protospacer is generally inserted nearest to the leader. However, sometimes this process appears to be reversed and a low level of *de novo* spacers (<2%) were found with the PAM end of the protospacer inserted distal to the leader for spacers acquired from pMGB1 in *S. solfataricus* and STSV2 in *S. islandicus* [72,76]. This presumably reflects defective recognition of the protospacer, the leader and/or the first repeat by the adaptation Cas proteins.

### 5.5. Alternative Spacer Acquisition Mechanism

**Figure 5:**
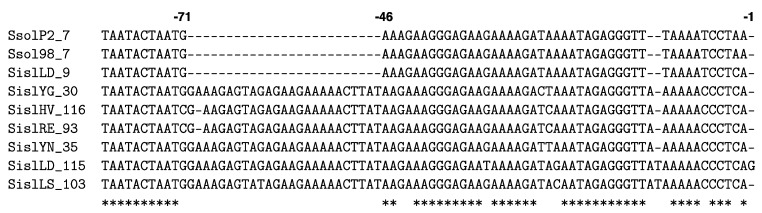
Alignment of locus E leaders from *S. solfataricus* strains P2 and 98/2 and *S. islandicus* strain LD.8.5 (SislLD) together with a selection of closely similar CRISPR leaders from different *S. islandicus* strains [55,87]. The number following the strain indicates the number of repeats in the CRISPR array. Position -1 lies adjacent to the first repeat. Conserved sequence positions are indicated by asterisks.

Locus E of *S. solfataricus* strains P1, P2 and 98/2 carries 6 spacers and the spacer and repeat sequences are identical, with the exception of the most leader-distal spacer of strain 98/2, and strain P3 carries four additional spacers (Figure 4) [42]. The mechanism of *de novo* spacer acquisition in locus E in strain P2 differed from that observed for the other active CRISPR loci in that spacers were inserted throughout the locus, albeit with a strong bias to repeat 4 (56%) and lesser bias to repeats 1, 3 and 6 (11%–17%). Moreover, only a single spacer insertion was observed for each clone [75]. An explanation for this apparent anomaly may lie in the sequence of the locus E leader which differs from those of other leaders in strain P2 and more closely resembles leaders of several *S. islandicus* strains, except that it carries a 25 bp deletion upstream from position -46 (Figure 5) [75,76]. Possibly, absence of this sequence impairs the specificity of the spacer insertion process.

A CRISPR locus with an almost identical leader region (one mismatch) and an identical repeat, but carrying seven different spacers, resides in *S. islandicus* L.D.8.5 on a highly conserved 36 kb genomic fragment with 99% sequence identity to the corresponding *S. solfataricus* P2 region. This strongly suggests the occurrence of an inter-genomic transfer despite the *S. islandicus* strain originating from Lassen National Park, USA and *S. solfataricus* P2 deriving from Naples, Italy [2,42,87].

### 5.6. Is Adaptation Activated by Interference?

While acquisition of *de novo* spacers can lead directly to interference in the Sulfolobales [72], for other organisms evidence has been presented for adaptation being induced by crRNA-directed interference, in both the genetically modified Type I-E system of *E. coli* [88,89] and in the Type I-B system of a haloarchaeon [90]. In addition, it was shown that the orientation of the matching spacer determines the genomic orientation of the protospacers selected for subsequent spacer acquisition; they are located in the same direction and at the PAM-distal end of the “priming” protospacer [88,89,90]. Such a feed-back mechanism has the intrinsic advantage of directing adaptation to the invading genetic element rather than the host chromosome, except when they carry similar sequence regions as occurs commonly, for example, with transposable elements of the Sulfolobales (Section 7.5).

No evidence was found for this mechanism operating in the Sulfolobales. For example, eight perfectly matching spacers in *S. solfataricus* did not induce spacer acquisition from the infecting SMV1 [75,76]. Nor were spacers acquired from SIRV2-infected *S. islandicus* LAL14/1, despite the presence of 21 SIRV2 matching host CRISPR spacers with five or less mismatches (Section 7.1) [91], and similarly, SIRV3 did not induce spacer acquisition despite the presence of closely matching spacers in *S. islandicus* REY15A [72]. Moreover, studies on adaptation in pMGB1 and STSV2 in the presence of SMV1, showed, with one exception (Section 5.3), that the protospacers selected during adaptation were randomly distributed with respect to both genome location and DNA strand [72,75].

Nevertheless, one cannot yet exclude that adaptation is stimulated by interference amongst the Sulfolobales. There are multiple partial, and some perfect, spacer matches to most known genetic elements present within each large CRISPR locus (Figure 4) [53,56] and it is possible that multiple spacer “priming” events occur simultaneously during adaptation, for a given genetic element, such that any protospacer orientation effects are masked.

### 5.7. The Conundrum of Reversible De Novo Spacer Acquisition

Experimental evidence was presented for reversion of *de novo* spacer acquisition in CRISPR loci of *S. islandicus* REY15A that was infected with the viruses SMV1 + STSV2 [72]. A single clone isolated 12 days p.i. had acquired single STSV2-derived *de novo* spacers in each of two CRISPR loci and STSV2 had been purged from the culture but SMV1 was still present. When this clone was subsequently cultured from a glycerol stock maintained at −80 °C, the culture was resistant to STSV2 infection but cell growth was strongly retarded and, unexpectedly, multiple SMV1-derived spacers were inserted in both CRISPR loci within two days, presumably induced by the cold-shock stress [72]. The *de novo* SMV1 spacers and the SMV1 content of the culture were then monitored over a 27 day period. Whereas SMV1 continued to propagate, despite the presence of perfectly matching *de novo* spacers, after 21 days all the *de novo* SMV1 spacers were lost from the culture. In addition, at 27 days, when SMV1 was still detectable in the culture by PCR, the single *de novo* STSV2 spacers were also lost and the CRISPR loci had reverted to their wild-type spacer contents. These changes were seen on different single clones obtained from the culture. Furthermore, during the 27 day period in which SMV1 spacers were acquired, and subsequently lost together with the STSV2 spacers, growth of the culture was strongly retarded [72].

The progressive loss of single *de novo* spacers most likely reflects a reversion of the Cas1-catalysed spacer insertion mechanism. Moreover, such a mechanism could facilitate reducing the extreme *de novo* spacer diversity generated during adaptation reactions [75], after the genetic element has been purged from the cell population. A similar mechanism could also have operated in the experiment where a single CRISPR spacer was challenged by a plasmid carrying a matching sequence and maintained under selection in *S. islandicus* [78] when half of the surviving transformants tested had specifically lost the single matching spacer.

An alternative explanation for the *de novo* spacer loss, that remains speculative, is that SMV1 integrated into the host chromosome and was then targeted by the SMV1-matching spacers such that cells carrying integrated SMV1 and matching *de novo* SMV1 spacers were destroyed. However, this would not explain why the STSV2 spacers were also subsequently lost from the STSV2-free culture [72]. Clearly, to understand this phenomenon one needs more information about how Cas1 functions during adaptation.

## 6. Molecular Mechanisms of Interference

### 6.1. Functional Significance of the Strand-Specificity of Spacer Matches

Early bioinformatical analyses of host CRISPR spacer sequence matches to viruses and plasmids of the Sulfolobales revealed many significant matches on both DNA strands of predicted open reading frames, consistent with interference targets being dsDNA [29,37,53]. In retrospect, since most *Sulfolobus* species carry Type I and multiple Type III interference complexes, these observed effects probably reflect that all spacer-derived crRNAs are available for Type I systems targeting dsDNA whereas only a fraction are utilised by Type III systems targeting transcripts or transcribing DNA, after undergoing additional processing at their 3'-ends [43,48,62,92] (Section 6.5).

### 6.2. Fidelity of crRNA-Spacer Recognition

Seminal studies on interference by the Type II system of *S. thermophilus* suggested that a single mismatch in crRNA-protospacer base pairing was sufficient to eliminate an immune response [93,94,95]. In contrast, studies on *Sulfolobus* indicated that a lower level of sequence matching was required, and that several mismatches could be tolerated with no strong reduction in Type I-A DNA interference in either *S. solfataricus* or *S. islandicus* [9,78].

A more recent study on *S. solfataricus* has reinforced this relatively low level of matching stringency but also provided evidence for the relative importance of the protospacer-matching sequence towards the 5'-end of crRNAs [96]. A conserved “seed” sequence was identified earlier within an eight nucleotide protospacer-matching sequence in this crRNA region, utilised by the *E. coli* Type I-E system [97]. However in *S. islandicus* the important crRNA annealing region is smaller, encompassing about five base pairs and the required matching less stringent [98]. Moreover, evidence was found for a second important annealing site towards the 3'-end of the crRNA [98]. For the two different Type III-B systems of *S. islandicus,* there was also a low stringency of crRNA-protospacer interactions but a specific sequence match at crRNA positions 28–30 was critical for effective interference [48].

The relatively low stringency required for crRNA-protospacer annealing during DNA and RNA interference increases the likelihood of fortuitous targeting of the host chromosome, a possibility that would be further reinforced by the large numbers of spacers present in most of the Sulfolobales.

### 6.3. Unequal Assembly of crRNAs into Interference Complexes

Indirect evidence for a range of crRNA effectivities was provided for Type III-D and Type III-B Cmr-β interference complexes of *S. solfataricus* P1 and P2, respectively [44,99], strains that carry genetic elements in an integrated form [21,100]. crRNAs were extracted from isolated Csm and Cmr complexes and deep sequencing revealed, for each complex, highly uneven crRNA yields distributed along CRISPR loci, with no bias to either end. Moreover, only a few crRNAs were present in high yield. The authors attribute the widely differing crRNA yields to inefficient processing of CRISPR transcripts owing to structural constraints and/or interference of CRISPR transcription by internal transcriptional signals [44,99].

There may be an additional explanation. It has been demonstrated that individual crRNAs from a given CRISPR locus can assemble in the structurally different Type I and Type III interference complexes, albeit with additional 3'-end processing for Type III interference [47,48]. Presumably, there are some structural constraints for an optimal interaction of the crRNA, with either complex, that extend beyond the 8 nt 5'-repeat tag and the sugar-phosphate backbone. It is likely that nucleotide sequence and strength of base-stacking will also influence the degree to which crRNAs assemble optimally into the different interference complexes.

### 6.4. Type I PAM-Dependent Interference

In Type I systems, interference is dependent, to some degree, on the PAM sequence. However, whereas different Type I-A subfamilies of *Sulfolobus* species rigorously identify specific PAM sequences during adaptation, most commonly either CCN or TCN with no significant bias for the third position [72], a few PAM-like sequences are recognised by the interference complex. In *S. islandicus* REY15A, for example, which uses the CCN motif for adaptation, the sequences CCN, CTN and TCN were susceptible to interference whereas GGN, GAN and TTN were inactive [78]. Moreover, for the Type I-B system of *Haloferax volcanii*, for which the PAM sequence was unknown, six triplets (ACT, TAA, TAT, TAG TTC and CAC) out of the 64 possible tested, were shown to be effective in interference [101]. These results suggested that the third nucleotide may play a role in interference in contrast to the results obtained for adaptation reactions [72,75,76]. Support for the latter supposition was presented for the Type I-A system of *Thermoproteus tenax*, which used a CCN PAM sequence for adaptation, where CCA and CCT produced strong interference but reduced activity was seen for TCA and none was detected for TCG [102].

The evidence for non-identical mechanisms of recognition of PAM sequences, and DNA strands, during adaptation and interference reactions led to the proposal to use the terms spacer acquisition motif (SAM) for adaptation and target interference motif (TIM) for interference [59].

### 6.5. Type III PAM-Independent Interference

At least two different archaeal Type III systems can mediate RNA interference via PAM-independent mechanisms employing crRNAs with additional processing at their 3'-ends. RNA silencing was first detected for a Type III-B system of the euryarchaeon *Pyrococcus furiosus* and evidence was presented for RNA cleavage, both *in vitro* and *in vivo*, employing a ruler mechanism on the target RNA, with no sequence cleavage specificity, and generating 3'-cyclic phosphate ends [43,92]. A Type III-B interference complex of *S. solfataricus* P2 carrying the non-core Cas protein Cmr7, that is exclusive to some of the Sulfolobales [47], was shown to cleave RNAs by a different mechanism, specifically cutting at U-A pairs yielding a 3'-OH and 5'-PO_4_ [44]. Consistent with the latter, genomes of the Sulfolobales are A-T-rich and only 11% of the spacers in *S. solfataricus* were shown to be devoid of A-T sequences. In addition, the guide crRNA was also cleaved but at a slower rate such that it could undergo limited recycling [44].

Later, using an SSV1-based genetic system, it was shown that similar Type III-B-directed mRNA cleavage could be induced in *S. solfataricus* strains *in vivo*, cutting at A-U or U-U sequences and, moreover, control experiments with the isolated Type III-B interference complex yielded similar degradation products, rendering it unlikely that the host Type III-D system had contributed to the observed *in vivo* interference [45].

This complexity of the interference mechanisms reached another level with the finding that one of two Type III-B complexes in *S. islandicus* REY15A targeted transcribing DNA *in vivo*, in combination with a non-core Cas protein Csx1 [47]. A similar specificity for transcribing DNA was subsequently demonstrated for a Type III-A system of *Staphylococcus epidermidis*, but no evidence was presented for the involvement of accessory Cas proteins [49]. The apparent contradiction with the demonstration that the Type III-A system from *S. thermophilus* targeted RNA, and not DNA [103] could possibly be reconciled if a dual RNA-DNA targeting mechanism operates as has been proposed for a *Sulfolobus* Type III-B-α system (see below).

Further studies have distinguished mechanistically between different Type III-B Cmr-α and Cmr-β interference modules operating in *S. islandicus* REY15A [48]. Structurally, the Cmr-β complex closely resembles the Type III-B complex of *S. solfataricus* described above with both carrying non-core Cmr7 proteins [44,47], and both complexes specifically target transcripts *in vivo* [45,48]. In contrast, the Cmr-α complex of *S. islandicus* targets both *de novo* transcripts and transcribing DNA together with the non-core Cas protein Csx1 [47,48]. This suggests that Cmr-β and Cmr-α complement one another’s interference mechanisms in *S. islandicus*, and that the Type III-B and Type III-D interference systems in *S. solfataricus* may cooperate similarly.

### 6.6. Quaternary Structures of Interference Complexes

**Figure 6:**
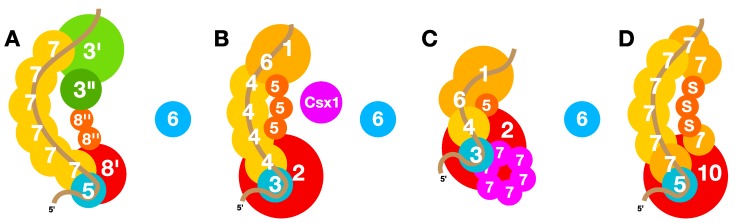
Schematic diagrams of CRISPR-Cas interference complexes. (**A**) A Type I-A complex where the Cas3' and Cas3", but not Cas6, are associated with the interference complex of the crenarchaeon *T. tenax* [102]. The crRNA is oriented as shown earlier for the genetically modified *E. coli* Type I-E complex [105]. (**B**) Type III-B Cmr-α complex of *S. islandicus* based on published structures of related complexes [46,106]. This complex requires Csx1 for targeting transcripts and transcribing DNA [47,48]. (**C**) RNA targeting Type III-B Cmr-β complex of *S. islandicus*, extrapolating from the Type III-B structure of *S. solfataricus* in [44]. (**D**) A Type III-D complex of *S. solfataricus* [99]. Estimated binding regions of crRNAs are colour-coded brown. Subunits of the Type I-A and III-D complexes are assigned Cas protein numbers while Type III-B complexes are given Cmr protein numbers. In (C) 7 denotes non-core protein Cmr7 which forms a pseudo-hexameric structure in the Sulfolobales. In (A) the protein locations indicated for Cas3'/3'' are speculative, while the putative position of a Cas8'' dimer is deduced from the published structure of a Type I-E interference complex [105].

Type I and Type III interference modules show major differences in their protein contents although some proteins are likely to be distant homologs [35,36,104]. Furthermore, their quaternary structures are distinct and diverse; a seahorse-like structure was first reported for the CASCADE complexes of *E. coli* Type I-E systems [105], whereas a low-resolution structure of an *S. solfataricus* Type III-B complex more closely resembled a “crab claw” [44]. The availability of higher resolution structures of additional Type I and III complexes suggest that they share a common central form that reconciles differences between the seahorse and crab-claw structures [46,99,105]. A schematic view of the most common *Sulfolobus* interference complexes based on results published for related systems is presented in Figure 6A–D.

Structures of the disparate Type III complexes have been shown to be remarkably similar [46,99,106,107] and there is no current evidence to suggest that the *S. islandicus* Cmr-α complex radically is different from other published structures of Type III-B complexes [46,106]. On the other hand, the Cmr-β complex, with the atypical crab claw-like structure, exhibits an unusual stoichiometry carrying only a single Cmr4 subunit and the non-core Cmr7 which forms a pseudo-hexameric structure (Figure 6B,C) [44].

Although no structure of a complete Sulfolobales Type I-A interference complex has been determined, protein association experiments have shown that hallmark characteristics such as the Cas7 backbone and Cas5/Cas8 base structure are conserved [62,102]. However, in contrast to the structures of the bacterial Type I-E and I-F complexes, both the Cas3' helicase and the Cas3'' nuclease are integral components of the complex, whereas the processing enzyme Cas6 is not. This is also in agreement with the genetic organisation of archaeal Type I-A cassettes, where *cas3'* and *cas3''* are invariably in operons with the other subunits, while *cas6* is often transcribed separately, consistent with the crRNA processing function of Cas6, and the crRNAs, being shared by different types of interference complexes within a host [70].

## 7. Inhibitory and Regulatory Mechanisms of Adaptation and Interference

### 7.1. Differential Regulation of Adaptation and Interference

An analysis of 190 archaeal CRISPR-Cas systems from 159 completed archaeal genomes in May 2013 identified 135 putative regulatory proteins with no apparent affinity to any specific type of CRISPR-Cas system [36]. Whereas some gene cassettes encoded no known regulatory proteins for others, including those of the Sulfolobales, separate Csa3 proteins were encoded in the adaptation and interference gene cassettes [42]. Putative Cas regulatory proteins are also encoded distantly from *cas* gene cassettes, including the *csa3* homolog ST1161 of *S. tokodaii,* suggesting that regulatory mechanisms are diverse. Structure determination of a Csa3 protein from *S. solfataricus* revealed a winged helix-turn-helix domain predicted to be involved in DNA recognition coupled to a putative ligand binding domain suggesting that its regulatory function may be dependent on specific ligands [108].

The role of Csa3 in regulating expression from the adaptation gene cassette was demonstrated recently in a genetic study in *S. islandicus* REY15A [109]. The protein was shown to bind to the promoter regions of the *csa1* and *cas1* genes and overexpression of Csa3 led to increased transcription of the adaptation gene cassette, increased Cas protein levels, and to hyperactive uptake of *de novo* spacers in host CRISPR loci, primarily but not exclusively from the over-expression vector.

Recent transcriptome studies on virus-infected *S. islandicus* species reinforce the independent, and differential, regulation of the adaptation and diverse interference modules. For example, on infecting *S. islandicus* LAL14/1 with SIRV2, transcription from the Type I-A and I-D interference gene cassettes was strongly enhanced, whereas the complementary adaptation gene cassettes remained silent throughout the infection period. Moreover, whereas Type III-B Cmr-α and Cmr-β gene cassettes were moderately expressed in uninfected cells, on SIRV2 infection the former was repressed and the latter enhanced [91]. The inactivity of the adaptation gene cassette is consistent with the observed absence of spacer acquisition and, moreover, this lack of activity indicates that there is no priming of adaptation by any of the 21 host CRISPR spacers with five or less mismatches to SIRV2 (Section 5.6).

In a separate study on STSV2-infected *S. islandicus* REY15A that was actively undergoing spacer acquisition, transcription from the adaptation gene cassette was enhanced on viral infection and strong transcriptional activation of the Type I-A interference gene cassette occurred but expression from both Type III-B Cmr-α and Cmr-β gene cassettes was repressed [110].

The observation that Cas modules, and particularly those of Type III-B, are regulated differently as a function of the infecting agent, the medium, and the progression of viral infection, suggests that the disparate classes of CRISPR interference modules have specific functions and are optimized, and regulated, for particular functions which we are only beginning to understand [110].

Tight regulation of CRISPR-Cas modules is often pronounced in bacterial CRISPR-Cas systems. For example, subtypes I-E and I-F are encoded in single operons and regulated by the nucleoid structuring H-NS protein and the LeuO transcription factor in *E. coli* [111,112]. Moreover, the diverse CRISPR-Cas systems of *Synechocystis* carry several putative regulators [113]. Thus CRISPR-Cas regulatory mechanisms are diverse and complex and may be especially important for minimising self-genome interference [77].

### 7.2. Inactivation of a Regulatory cas Gene by Genetic Element Integration

**Figure 7:**
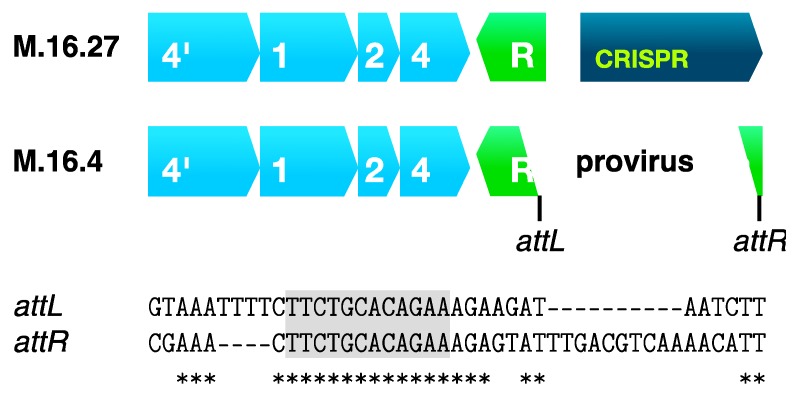
Adaptation *cas* gene cassette of *S. islandicus* M.16.4 where the *csa3* gene encoding a putative transcriptional regulator is interrupted by an integrated plasmid or virus via the predicted integration *att* sites that are shown (modified from [56]). The closely related strain M.16.27 lacks the integrated element [87].

The genome of *S. islandicus* M.16.4 isolated from the Mutnovsky Volcano, Kamchatka, Russia carries a *csa3* gene physically linked to an adaptation gene cassette [87] and the Csa3 protein is likely to regulate transcription from the gene cassette [38]. However, in this strain the *csa3* gene carries an integrated genetic element of viral or plasmid origin that had apparently integrated at the indicated *att* site (Figure 7) [56]. The sequence of the interrupted *csa3* gene is highly conserved, relative to those of related strains, suggesting that the integration event is reversible [56]. Another strain M.16.27, from the same solfataric field, exhibits an intact *csa3* gene and, unlike strain M.16.4, it carries a CRISPR spacer with a perfect sequence match to the integrated element [56,87]. Thus, the integrated genetic element in strain M.16.4 can potentially inhibit expression of the adaptation Cas proteins and, as demonstrated for another *S.* *islandicus* strain [109], this will prevent expression of Csa3 and thereby inhibit spacer acquisition. Consequently, other copies of the genetic element will be able to propagate in strain M.16.4, whereas for strain M.16.27, the CRISPR Type I-A interference system could eliminate the invading genetic element.

### 7.3. CRISPR-Cas Interference Avoidance and Anti-CRISPR Systems

The relatively low stringency of sequence matching required for crRNA-protospacer annealing during Type I-A interference in *Sulfolobus* species suggests that minor mutations in targeted protospacers are unlikely to be effective in preventing interference (Section 6.2). However, when the *Sulfolobus* rudivirus SIRV1 was passed through different Icelandic *S. islandicus* hosts, including strain LAL14/1 that carries SIRV1 matching spacers [58], major changes occurred in the viral genome including rearrangements, deletions and extensive changes in gene sequences [114]. Multiple 12 bp indels also occurred, generally within ORFs, which were later shown to be a common feature of rudiviruses and lipothrixviruses [114,115]. In a related experiment, a thermoneutrophilic environmental sample was maintained in a bioreactor over two years, and extensive gene and genomic differences were detected in multiple variants of a hyperthermophilic archaeal virus HAV1, possibly resulting from CRISPR-Cas-directed interference in unidentified hosts [116]. Some of the genomic rearrangements were predicted to have arisen at specific recombination sites distributed along the linear HAV1 genome [116] by a similar mechanism to that observed for the archaeal conjugative plasmids (Section 2.2).

Viruses of the different Sulfolobales families (Table 1) show considerable diversity in their gene contents. This may arise in different ways, including the modular exchange observed for lipothrixviruses [117], and the putative mechanism for generating hybrid viruses [118] (Section 7.4). Thus, the above results suggest that, in response to the relatively low stringency crRNA-protospacer sequence matching required for CRISPR-Cas interference, the Sulfolobales viruses and conjugative plasmids may have evolved specific mechanisms for generating genomic deletions, insertions and rearrangements.

Evidence has also been found for small diverse anti CRISPR-Cas proteins encoded by phages that infect *Pseudomonas aeruginosa* which enabled them to avoid CRISPR-Cas interference [119,120]. In some *Sulfolobus* species, SMV1 was resistant to CRISPR-Cas interference despite the presence of perfectly matching spacers but, in one experiment, it eventually lost its immunity coincident with the loss of a small, DNA binding, virion protein. The latter could, potentially, constitute an anti-CRISPR protein that specifically protected SMV1 [72].

### 7.4. Integration and Interference

Archaeal genetic elements integrate by at least two different mechanisms, one of which is archaea-specific and involves partitioning of the integrase gene on insertion, generally within tRNA genes [121,122]. A possible consequence of this mechanism is that genetic elements become trapped in host genomes when cells are cured of the free genetic element and no functional integrase is produced for excision [123]. Since CRISPR-Cas systems tend to eliminate free forms of genetic elements they may also tend to enhance genomic entrapment of the individual integrative elements.

However, this archaeal integration mechanism also provides a possible means for generating variants of certain viruses with integrative circular dsDNA genomes which could avoid CRISPR interference. Redder *et al.* [118] proposed a model whereby circular dsDNAs from similar fuselloviruses, with partitioning integrase genes, might integrate at the same tRNA *att* site and undergo recombination at different sites, with the subsequent release of diverse hybrid viral genomes, each carrying an intact integrase gene. At present, this is speculative but, in principle, it could provide a mode of viral diversification to avoid CRISPR-Cas interference if the excised variant viral DNAs have lost spacer-matching sequences.

It remains unclear whether and how Type I systems distinguish mechanistically between free and integrated forms of genetic elements unless, for example, expression of the interference Cas proteins is down-regulated on integration. However, examination of regions of Sulfolobales genomes bordering tRNA genes frequently reveal a few adjacent genes of viral or plasmid origin that are fragmented or otherwise degenerate [55,124]. Possibly the extensive fragmentation of integrated genetic elements seen in these genomes arises, at least partially, as a result of CRISPR-Cas interference and subsequent DNA repair.

### 7.5. CRISPR-Cas Defence and Transposition

Most Sulfolobales, with the notable exception of *S. acidocaldarius* [30], are very rich in transposable elements including IS elements and non-autonomous MITEs [79,81] and some of these transposons are encoded by archaeal viruses and plasmids [76,125]. Transcriptome analyses have shown that increased transpositional activity commonly occurs on infection with *Sulfolobus* viruses including STIV [126], SIRV2 [91,127] and STSV2 [110].

In two experiments, this transpositional activity could be directly linked to the CRISPR-Cas response during both adaptation and interference reactions. One *orfB* element (IS605 family) was shown to be gradually lost from the conjugative plasmid pMGB1 propagating in *S. solfataricus*, during the time period when pMGB1 was undergoing adaptation [76]. Moreover, the IS element ISC1359 was shown to insert into, and inactivate, a spacer encoding a crRNA that matched a plasmid maintained under selection [78]. These results suggest that transpositional events can be specifically activated by CRISPR-Cas defence. IS elements have also been implicated in producing genomic deletions and rearrangements of CRISPR-Cas systems in *S. solfataricus* and in *E. coli* [40,82].

### 7.6. Antisense CRISPR RNAs Can Impair Interference Effects

*Sulfolobus* species produce large numbers of non-coding RNAs (ncRNA) that have been implicated in various functions including RNA modification, and regulation of both gene expression and transpositional activity [61,69,128]. For *S. solfataricus* P1 and P2, about 60 different RNAs have been isolated and sequenced, and a transcriptome study of the latter strain provided evidence for, potentially, a further 232 ncRNAs, in addition to those produced from processing of CRISPR transcripts [69].

Reverse transcripts were originally detected from CRISPR loci of *S. acidocaldarius* by Northern blotting [29,37] and, later, transcriptional start sites and levels of antisense CRISPR RNAs could be quantified from total transcriptome data obtained for *S. solfataricus* P2 [69]. A likely explanation for the antisense CRISPR RNA transcripts, apart from read-through into the leader-distal end of CRISPR loci [29], is that they initiate and end in protospacers carrying promoter or terminator motifs, respectively, that are taken up from invading genetic elements. In support of this, many potential promoter and terminator motifs were identified in CRISPR loci of the Sulfolobales [70]. Similarly, internal transcription signals will also occur in the sense direction that can potentially interfere with, or reinforce, primary transcription along CRISPR loci [70].

An early hypothesis was that antisense RNAs might participate in a eukaryal-like RNAi defence [129]. To test this hypothesis, attempts were made to isolate dsRNA complexes of crRNAs and antisense CRISPR RNAs from *S. acidocaldarius*, which carries Type I-A, Type III-D and a variant CRISPR-Cas system [36], but they all failed [37]. A possible reason for this failure became clearer later when it was shown for *P.* *furiosus* that an antisense CRISPR transcript was targeted and cleaved *in vivo* by a Type III-B interference complex [92]. Thus, antisense CRISPR RNAs are potential targets for Type III-directed RNA interference which, in turn, has implications for the levels of functional crRNAs because they will also be cleaved during antisense RNA interference [47,92]. However, targeting of antisense CRISPR transcripts by Type III-B Cmr-α interference complexes [47] would not lead to cleavage of a transcriptionally active region of the CRISPR locus as long as the antisense RNA sequence is present that perfectly matches the 5 or 8 nt 5'-repeat tag of the crRNA.

## 8. Role for Toxins

CRISPR loci and the adaptation and interference gene cassettes are located in large variable regions of *Sulfolobus* genomes which can extend up to about 400 kbp in size [55,125]. These regions generally carry multiple copies of diverse toxin-antitoxin gene pairs, averaging about 24 pairs per genome [130]. Moreover, the regions are rich in transposable elements and at least some rearrangements in these regions occur via recombination events between similar mobile elements [79,81].

A possible functional link with CRISPR-Cas defence systems was highlighted by the observation that *vapBC* toxin-antitoxin gene pairs are often interspersed in *cas* gene cassettes, sometimes in multiple copies. For example, a Type III-D gene cassette of *Acidianus* *hospitalis* carries four different *vapBC* pairs and it was suggested that some of the toxin–antitoxin pairs may facilitate maintenance of the CRISPR-Cas systems in the host population [39,125].

Toxins are also implicated in the strong retardation of cell growth that occurs during adaptation [72,75] and it has been proposed that some Cas proteins may be toxins; Cas2 proteins, in particular, can exhibit RNAse or DNAse activity, characteristic of toxins [103,131]. The first experimental evidence implicating Cas proteins in toxin activity arose from a genetic study of the Csa5 protein (SSO1443) encoded in a Type I-A interference gene cassette of *S. solfataricus*. Overexpression of the protein was shown to kill *S. solfataricus* cells and infection with the rudivirus SIRV2 induced Csa5 expression to a level that was toxic for the cells [132].

## 9. Functional Importance of Non-Core Cas Proteins

Many protein genes, in addition to those of toxins–antitoxins, are found interwoven in *cas* gene cassettes and especially those of the Type-III interference modules. The physical proximity of these genes is suggestive that they cofunction with CRISPR-Cas systems [36,39]. Support for a functional link to CRISPR-Cas systems is provided by the finding that some of the proteins show evidence of having specifically co-evolved with core Cas proteins [36]. These non-core Cas proteins include diverse members of the Csx1 superfamily, different ATPases, transcriptional regulators, proteases, helicases, nucleases, and small RRM domain-containing proteins, all which have been summarised recently for all archaea [36].

The non-core *cas* genes are often found associated with Type III interference gene cassettes in different combinations. For example, in the genomes of *S. tokodaii* and *S. islandicus* similar *csx1* genes are associated with the different subtype III-A, III-B and III-D interference complexes [47] and, conversely, in other archaeal genomes highly similar Type III interference gene cassettes are often associated with different sets of non-core *cas* genes, or lack them, suggesting that these genes can exchange between different Type III gene cassettes and influence them functionally [36]. Major classes of non-core Cas proteins associated with Type III systems are summarised below.

### 9.1. Csx1 Superfamily Proteins

The Csx1 superfamily constitutes the largest group of non-core Cas proteins. The proteins share an N-terminal domain [36], recently named the CARF (CRISPR-Cas Associated Rossmann Fold) domain [50] which may interface with interference complexes. In contrast, the C-terminal domains are very diverse structurally and are predicted to exhibit DNA binding and diverse nuclease activities [50]. Direct support for their potential functional importance arose when a Csx1 protein was shown to be important for Cmr-α-directed interference of actively transcribing DNA in *S. islandicus* [47] (Section 6.5).

### 9.2. Proteases

Proteases comprise another class of accessory Cas proteins and the Sulfolobales-specific variant Type III system exhibits a putative aspartate protease encoded within the interference gene cassette (Figure 1B). Although the role of this protease in interference activity remains unclear, it may be involved in the maturation of protein subunits during assembly of the interference complex.

### 9.3. ATPases

ATPases are common accessory Cas proteins and their genes are often cotranscribed with additional non-core Cas genes encoding small RRM domain proteins (e.g., the YN1551_2137/38 gene pair adjacent to an *S. islandicus* Type III-A system) [36]. They may generate energy for catalysing interference activity. For example, Type I systems require energy for unwinding target DNA generated by the Cas3 helicase, but Type III systems do not employ a core Cas helicase. Although a helicase may be unnecessary for mRNA interference an ATPase could be important, for example, when transcribing DNA is a target as proposed for the *S. islandicus* Cmr-α complex [48,70]. In addition, ATPases could facilitate cleavage reactions that are thermodynamically unfavourable, for example, when levels of target RNAs are low [44].

### 9.4. CRISPR Repeat Binding Proteins Cbp1 and Cbp2

A search for CRISPR repeat binding proteins amongst *Sulfolobus* species yielded a protein, designated Cbp1 (CRISPR repeat binding protein), carrying three imperfect repeats interspaced with basic linker regions that is encoded by several *Sulfolobus* species [70,133]. Although no evidence has been found for a universal archaeal CRISPR repeat-binding protein, a related protein, Cbp2, was found encoded by several archaeal thermoneutrophiles carrying two imperfect repeats joined by a single linker. An NMR analysis of Cbp2 yielded a homeodomain-like structure with each repeat forming a helix-turn-helix domain [134]. Genetic experiments showed that when the *cbp1* gene was deleted, a strong reduction in pre-crRNA yields occurred and when overexpressed pre-crRNA yields increased. It was concluded, therefore, that Cbp1 minimizes interference from transcriptional signals carried on spacers deriving from the A-T-rich genetic elements, and on some CRISPR repeats [70]. Neither Cbp1 nor Cbp2 are linked genomically to CRISPR loci or Cas genes suggesting that they have additional cellular functions, probably also related to transcriptional regulation.

## 10. Conclusions

A number of questions have been raised that specifically relate to CRISPR-Cas systems of the Sulfolobales. They include: (1) why are the cellular CRISPR loci so extensive, tending to be both large and present in multiple copies, (2) why, despite the relatively low stringency of crRNA-protospacer annealing, are so many matching spacers maintained in the CRISPR loci against a given type of virus or conjugative plasmid, and (3) why, despite their carrying multiple CRISPR-Cas systems and numerous matching spacers (Figure 4), are *S. solfataricus* strains P1 and P2 such good laboratory hosts for many diverse viruses and conjugative plasmids [2]?

We infer that the three questions are related and that a likely explanation is that the CRISPR-Cas systems of the Sulfolobales function relatively inefficiently during interference. Thus, many mature crRNAs may not be formed because of incomplete primary transcript elongation or defective processing. Moreover, some crRNAs may exhibit features that impede optimal assembly into one or more interference complexes, including specific sequences or secondary structures or strong base stacking. In addition, different interference complexes will compete for the same crRNAs. This hypothesis receives some support from the demonstration that the crRNA contents of the Type III-D and Type III-B Cmr-β interference complexes of *S. solfataricus* were strongly biased to a few spacers randomly distributed along the CRISPR loci [44,99]. Furthermore, targeting of antisense RNAs could lead to a depletion of the complementary crRNAs [92] (Section 7.6). In addition, the archaeal viruses and conjugative plasmids appear to have evolved effective mutational mechanisms for avoiding CRISPR-Cas interference (Section 7.3).

Although this argument for inefficient interference sounds counter-intuitive, it could explain the successful use of *S. solfataricus* strains P1 and P2 as laboratory hosts for diverse genetic elements [2], in that although their copy numbers may be reduced to a low level by CRISPR-Cas interference, they can still propagate. A further advantage of inefficient interference via multiple crRNAs is that it would obviate the need for activating the energy-demanding adaptation process that is coincident with severe growth retardation of the adapting cells over many days [72,75].

There is also a regulatory aspect that contributes to the apparent complexity of the Sulfolobales CRISPR-Cas systems. Although CRISPR transcripts appear to be produced constitutively in the absence of infecting genetic elements [29,37], *cas* gene cassettes for adaptation and interference are individually and differentially regulated, and the regulation is influenced by invading genetic elements, and other factors [67,72,110]. Moreover, *S. solfataricus* carries two Type I-A subfamilies (with CCN and TCN PAM sequences) that are associated with different sets of CRISPR loci, and they are regulated separately, and differently, during both adaptation [75,76] and interference [44,99].

A final question relates to why multiple, different, Type III interference systems often coexist intracellularly in the Sulfolobales. Current work indicates that they are functionally diverse, targeting either transcripts, or the RNA and DNA of actively transcribing regions [45,47,48]. Moreover, the non-core Cas protein Csx1 has been implicated in interference [47] and given the large number and variety of non-core Cas proteins that appear to have coevolved with Type III interference Cas proteins [36], it is likely that a variety of different interference mechanisms can operate intracellularly.
